# The PH domain from the *Toxoplasma gondii* PH-containing protein-1 (TgPH1) serves as an ectopic reporter of phosphatidylinositol 3-phosphate in mammalian cells

**DOI:** 10.1371/journal.pone.0198454

**Published:** 2018-06-05

**Authors:** Krishna Chintaluri, Brady D. Goulden, Camilyn Celmenza, Golam Saffi, Emily Miraglia, Gerald R. V. Hammond, Roberto J. Botelho

**Affiliations:** 1 Department of Chemistry, Ryerson University, Toronto, Ontario, Canada; 2 The Molecular Science Graduate Program, Ryerson University, Toronto, Ontario, Canada; 3 Department of Cell Biology, University of Pittsburgh School of Medicine, Pittsburgh, Pennsylvania, United States of America; Queen Mary University of London, UNITED KINGDOM

## Abstract

Phosphoinositide (PtdInsP) lipids recruit effector proteins to membranes to mediate a variety of functions including signal transduction and membrane trafficking. Each PtdInsP binds to a specific set of effectors through characteristic protein domains such as the PH, FYVE and PX domains. Domains with high affinity for a single PtdInsP species are useful as probes to visualize the distribution and dynamics of that PtdInsP. The endolysosomal system is governed by two primary PtdInsPs: phosphatidylinositol 3-phosphate [PtdIns(3)P] and phosphatidylinositol 3,5-bisphosphate [PtdIns(3,5)P_2_], which are thought to localize and control early endosomes and lysosomes/late endosomes, respectively. While PtdIns(3)P has been analysed with mammalian-derived PX and FYVE domains, PtdIns(3,5)P_2_ indicators remain controversial. Thus, complementary probes against these PtdInsPs are needed, including those originating from non-mammalian proteins. Here, we characterized in mammalian cells the dynamics of the PH domain from PH-containing protein-1 from the parasite *Toxoplasma gondii* (TgPH1), which was previously shown to bind PtdIns(3,5)P_2_ *in vitro*. However, we show that TgPH1 retains membrane-binding in PIKfyve-inhibited cells, suggesting that TgPH1 is not a viable PtdIns(3,5)P_2_ marker in mammalian cells. Instead, PtdIns(3)P depletion using pharmacological and enzyme-based assays dissociated TgPH1 from membranes. Indeed, TgPH1 co-localized with Rab5-positive early endosomes. In addition, TgPH1 co-localized and behaved similarly to the PX domain of p40phox and FYVE domain of EEA1, which are commonly used as PtdIns(3)P indicators. Collectively, TgPH1 offers a complementary reporter for PtdIns(3)P derived from a non-mammalian protein and that is distinct from commonly employed PX and FYVE domain-based probes.

## Introduction

Phosphoinositides (PtdInsPs) are signaling lipids that modulate many important cellular functions including signal transduction, cell proliferation, cell migration, and membrane trafficking. There are seven species of PtdInsPs based on the differential phosphorylation of the inositol headgroup that are embedded in the cytosolic leaflet of cellular membranes and act as docking sites for numerous protein effectors. Importantly, each PtdInsP species binds to a unique set of cognate effectors and displays a characteristic subcellular distribution. The spatial restriction of PtdInsPs coupled to the recruitment of species-specific PtdInsP effector proteins endows the host membrane or organelle with their attributable functions and identity [1–4]. Given these broad roles, dysregulation of PtdInsPs can drive many diseases such as cancer, diabetes, obesity and rare genetic disorders [3,5–7].

The subcellular distribution of PtdInsPs is partly informed through effector proteins and their inositol-lipid binding domains [4,8,9]. A diverse number of PtdInsP-interacting protein domains have been discovered and include the ENTH, GRAM, FYVE, PH, PHD, and PX domains, among others [4,9]. The affinity and specificity of these domains towards each PtdInsP can differ greatly; some domains exhibit superb specificity and high affinity for a single PtdInsP species, while others are promiscuous and/or exhibit low affinity [9–12]. Importantly, chimeras of fluorescent proteins and PtdInsP-binding domains that show high specificity and affinity for a single PtdInsP species can reveal the localization of the target PtdInsP via fluorescence microscopy [8,13–17]. There is now a large array of widely employed PtdInsP reporters based on this strategy [8,9,18]. However, these protein domain-based probes are not without caveats since they may also interact with additional endogenous ligands, thus preferentially detecting PtdInsP pools that co-exist with the additional ligand [19–25]. Thus, there is a need for additional complementary PtdInsP reporters.

The endolysosome system is regulated by two primary PtdInsPs: PtdIns(3)P and PtdIns(3,5)P_2_. PtdIns(3)P is synthesized by Vps34—a class III PI 3-kinase—and governs early endosome fusion, maturation and cargo sorting [15,22,23,26,27]. Using the PX domain from p40^phox^ and the FYVE domains from Hrs and EEA1 as probes, PtdIns(3)P is thought to primarily localize to early endosomes, though it is also transiently detectable in phagosomes and omegasomes, which are precursors to autophagosomes [15,27–30]. PtdIns(3)P is terminated in part by PIKfyve-mediated conversion to PtdIns(3,5)P_2_ [31]. Disruption of PtdIns(3,5)P_2_ leads to various defects, but most striking of all is the massive enlargement of endolysosomes, suggesting that it functions at the level of these organelles [31–37]. A tandem fusion of the N-terminal of TRPML1, a lysosomal Ca^2+^ channel regulated by phosphatidylinositol 3,5-bisphosphate [PtdIns(3,5)P_2_], may report the localization of this lipid [38,39]. Nevertheless, the specificity of this probe towards PtdIns(3,5)P_2_ was questioned in at least some cell types [40].

More recently, the PH domain of PH-containing protein-1 found in *Toxoplasma gondii* (TgPH1) was reported to have specificity towards PtdIns(3,5)P_2_ in that parasite [41]. TgPH1 was isolated during affinity precipitation with PtdIns(3,5)P_2_-beads from *T*. *gondii* lysates and shown to interact with PtdIns(3,5)P_2_ and PtdIns(3)P using in vitro assays [41]. Here, we generated constructs to express GFP-fusion of TgPH1 and evaluated its suitability as a PtdIns(3,5)P_2_ probe in mammalian cells. However, using pharmacological inhibitors and a genetically encoded system to deplete PtdInsPs, we provide evidence that TgPH1 reports PtdIns(3)P, not PtdIns(3,5)P_2_, in mammalian cells. Thus, TgPH1 expands the molecular toolbox to investigate PtdIns(3)P by offering a non-mammalian derived protein domain probe distinct from the FYVE and PX domains that are typically employed to study this lipid.

## Materials and methods

### Nucleic acids

Plasmids encoding 2FYVE-RFP and p40PX-mCherry were kindly provided by Dr. Sergio Grinstein. LAMP1-mRFP, mCherry-Rab5 and mCherry-FYVE were kindly provided by Dr. Tamas Balla. GFP-PIKfyve, GFP-PIKfyve^K1831E^ were a generous gift from Dr. Assia Shisheva. iRFP-FRB-Rab5, mCherry-FKBP-INPP5E, mCherry-FKBP-MTM1 and mCherry-FKBP-MTM1^C375S^ were previously characterized [42–44]. We generated various constructs encoding fluorescent TgPH1 fusion proteins including GFP-TgPH1, GFP-2x-TgPH1, eGFP^NES^-TgPH1 and NES-iRFP-TgPH1 as follows: GFP-TgPH1 and GFP-2xTgPH1 constructs were synthesized in pcDNA3.1::N-eGFP backbone (Genscript, Piscataway, NJ). For pcDNA3.1::N-eGFP-2x-TgPH1, a GSGN linker was inserted between the two tandem copies of TgPH1. The sequence of TgPH1 (toxodb.org: TGGT1_260370) was synthesized into the pcDNA 3.1::N-eGFP vectors using the KpnI and NotI sites. The EGFP^NES^-TgPH1 was constructed into a pEGFP-C1 vector (Clontech, Mountain View, CA), incorporating the nuclear export sequence from *X*. *laevis* MAPKK1 cloned in frame with the 5’ of eGFP start codon to reduce translocation of GFP-fusion proteins into the nucleus. NES-iRFP-TgPH1 was built using pEGFP-C1 backbone, replacing EGFP with iRFP713. Plasmids were prepared with an endonuclease-free midi-preparation plasmid kit (VWR, Mississauga, ON) according to manufacturer’s instructions.

### Cell culture and transfection

RAW 264.7 cells (ATCC TIB-71), HeLa cells (ATCC CCL-2), COS-7 cells (ATCC CRL-1651), PC3 cells (ATCC CRL-1435) were obtained from ATCC (ATCC, Manassas, VA). ARPE-1 (or RPE) cells were a kind gift from Dr. Costin Antonescu at Ryerson University. RAW and HeLa cells were maintained in 25 cm^2^ filter-cap flasks, while COS-7 cells were grown in 75 cm^2^ filter-cap flasks with Dulbecco’s modified Eagle’s medium (DMEM; ThermoFisher, Burlington, ON) supplemented with 10% heat-inactivated fetal bovine serum (FBS; ThermoFisher). PC3 cells were maintained in RPMI without phenol red (Gibco) and RPE cells were maintain in DMEM/F12 medium (ThermoFisher); in both cases, media were supplemented with 10% FBS, 1% L-glutamine (Gibco) and 1% penicillin/streptomycin. For COS-7 cells, the medium was additionally supplemented with 100 units/mL penicillin, 100 μg/ml streptomycin and 1:1000 chemically defined lipid supplement (ThermoFisher). Passaging of RAW cells was done by scraping, or using Trypsin-EDTA (0.25% Trypsin with EDTA; ThermoFisher) for the other cell types. For experiments with RAW, HeLa, RPE and PC3 cells, cells were seeded at ~25 to 30% confluency onto 12-mm square glass coverslips (VWR) or 18-mm circular glass coverslips (Electron Microscopy Sciences, Hatfield, PA). These cells were transfected for 24 h with 1 μg of plasmid DNA using FuGENE HD (Promega, Madison, WI) as per manufacturer’s instructions. For experiments with COS-7, cells were seeded at ~25% confluence on 35-mm dishes with 20-mm glass coverslip bottoms (CellVis, Mountain View, CA) coated with 10 μg/ml fibronectin. Cells were transfected for 18–28 h with 600 ng of plasmid encoding FKBP-conjugated phosphatase enzyme, 200 ng of plasmid encoding iRFP-FRB-Rab5 and 200 ng of plasmid encoding NES-eGFP-TgPH1 complexed with 3 μg Lipofectamine 2000 (ThermoFisher) for 20 min in 0.2 ml Opti-MEM (ThermoFisher).

### Pharmacological depletion of phosphoinositides

Unless otherwise stated, cells were treated with 20 nM apilimod (Toronto Research Chemicals, Toronto, ON) or with 100 nM YM201636 (AdooQ Biosciences, Irvine, CA) for 1 h to deplete PtdIns(3,5)P_2_, [45,46]. Alternatively, cells were exposed to Vps34-IN1 (Millipore Sigma, Toronto, ON) at 1 μM for 1 h to deplete PtdIns(3)P [26]. For inducible-phosphatase depletion of PtdInsPs, rapamycin was added to cells at a final concentration of 1 μM (see below).

### Live and fixed cell imaging

For live cell imaging, cells were pre-loaded with a 1.5 h pulse of 150 μg/mL fixable, anionic dextran conjugated to Alexa Fluor™ 546, 10,000 MW (ThermoFisher), followed by 1 h chase with fresh medium. Cells were then manipulated with pharmacological treatments as described above and then subjected to live-cell imaging. Imaging was performed at ambient CO_2_ with cells submerged in HEPES-buffered RPMI supplemented with 5% FBS. Imaging was performed by spinning disc confocal microscopy using Quorum DisKovery spinning disc confocal microscope system connected to an Andor Zyla 4.2 megapixel sCMOS camera and using a 63 X 1.4 NA oil-immersion objective (Quorum, Guelph, ON). Standard excitation and emission filter cubes and lasers were then employed.

For live-cell imaging of rapamycin-inducible PtdInsP depletion in COS-7 cells, media was exchanged for FluoroBrite DMEM (ThermoFisher) supplemented with 25 mM HEPES (pH 7.4), 10% FBS, 2 mM Glutamax (ThermoFisher) and 1:100 chemically-defined lipid supplement. Then, 1 μM rapamycin (ThermoFisher) was added by bath application just before imaging. Imaging was performed on a Nikon A1R laser-scanning confocal microscope operating in fast “resonant” mode with 8-frame accumulation, using a 100x 1.45 NA plan-apochromatic oil-immersion objective on a Nikon TiE inverted microscope stand. GFP and iRFP were acquired concurrently using 488 and 640 nm laser excitation and 475–525 nm and 662–738 nm bandpass filters, whereas mCherry signal was acquired on a separate scan using 561 nm excitation and a 565–615 nm bandpass filter to avoid cross-talk. A motorized stage was used to image up to ten separate fields at 30 s frame intervals.

### Image and statistical analysis

To quantify membrane-associated to cytosolic ratio of TgPH1 probes, we imported images into ImageJ, and then assigned 3 or 10-pixel wide lines measuring 20-40-pixels in length along areas of transfected cells that excluded the nucleus. Plot profiles were then obtained and exported into spreadsheet program, values were ordered according to fluorescent intensity and the ratio of the highest 10 pixels over the lowest 10 pixel values (F_H_/F_L_ fluorescence ratio) was calculated with the expectation that cytosol-distributed signal was produce values approximate to 1, while signal that accumulates in puncta relative to cytosol with produce values greater than 1. Alternatively, whole cell was thresholded to obtain punctate structures and the fluorescence intensity of these structures was quantified. We then took the average fluorescence of the entire cell and obtain a puncta to cytosol fluorescence F_p_/F_c_. All analyses were performed using at least 20 cells per condition per experiment, across at least three independent experiments. For each condition, averages and standard deviation (STD) or standard error of the mean (SEM) were calculated.

For the rapamycin-inducible depletion of PtdInsPs in COS-7 cells, the Rab5 signal was used to generate a binary mask. The fluorescence intensity of TgPH1 under the Rab5 mask was then measured relative to the whole cell as described previously [42]. The means were subject to standard error of the mean taken from 41 (MTM1), 23 (C375S) or 27 (INPP5E) cells from three or four independent experiments.

All data were then subjected to statistical analysis. We used Student’s t-test when comparing only two conditions or ANOVA, followed by the Tukey’s post-hoc test when comparing more than two conditions.

## Results

### TgPH1 binds to intracellular membranes independently of PtdIns(3,5)P_2_ in mammalian cells

TgPH1 was shown to bind PtdIns(3,5)P_2_ using *in vitro* affinity precipitation with PtdIns(3,5)P_2_-beads, liposomes and lipid blots, though the lipid blot also showed significant binding to PtdIns(3)P [41]. Thus, we speculated that TgPH1 could serve as a biosensor for PtdIns(3,5)P_2_ in mammalian cells. To assess this, we generated various mammalian expression vectors encoding fluorescent chimeras of single TgPH1 domain (GFP-TgPH1, GFP^NES^-TgPH1, iRFP^NES^-TgPH1) and tandem TgPH1 domains (GFP-2x-TgPH1); the GFP^NES^ and iRFP^NES^ were engineered to include a nuclear export sequence (NES) to reduce the tendency of some fluorescent proteins to accumulate in the nucleus. We started our analysis by transfecting and comparing GFP, GFP-TgPH1 and GFP-2x-TgPH1 in RAW macrophages, which possess a very dynamic endolysosomal membrane trafficking system. RAW cells were labelled with fluorescent dextran to demarcate lysosomes, followed by treatment with DMSO-only or 20 nM apilimod or 100 nM YM201636 for 1 h to inhibit PIKfyve activity [45,46]. These compounds were previously shown to deplete >80% of PtdIns(3,5)P_2_ in various cells such as RAW macrophages [45–47]. To estimate the extent of membrane association, we calculated the average fluorescence ratio of the 30% most intense pixels over the 30% least intense pixels (F^H^/F_L_) cell. We did this by drawing 10-pixel thick lines that crossed randomly selected regions (when no obvious puncta were available to score), puncta and vesicles, i.e., structures with unresolved lumen, or using a 3-pixel thick line that abutted the limiting membrane of vacuoles in parallel to the vacuolar diameter to avoid the “dark” lumen.

**Fig 1:**
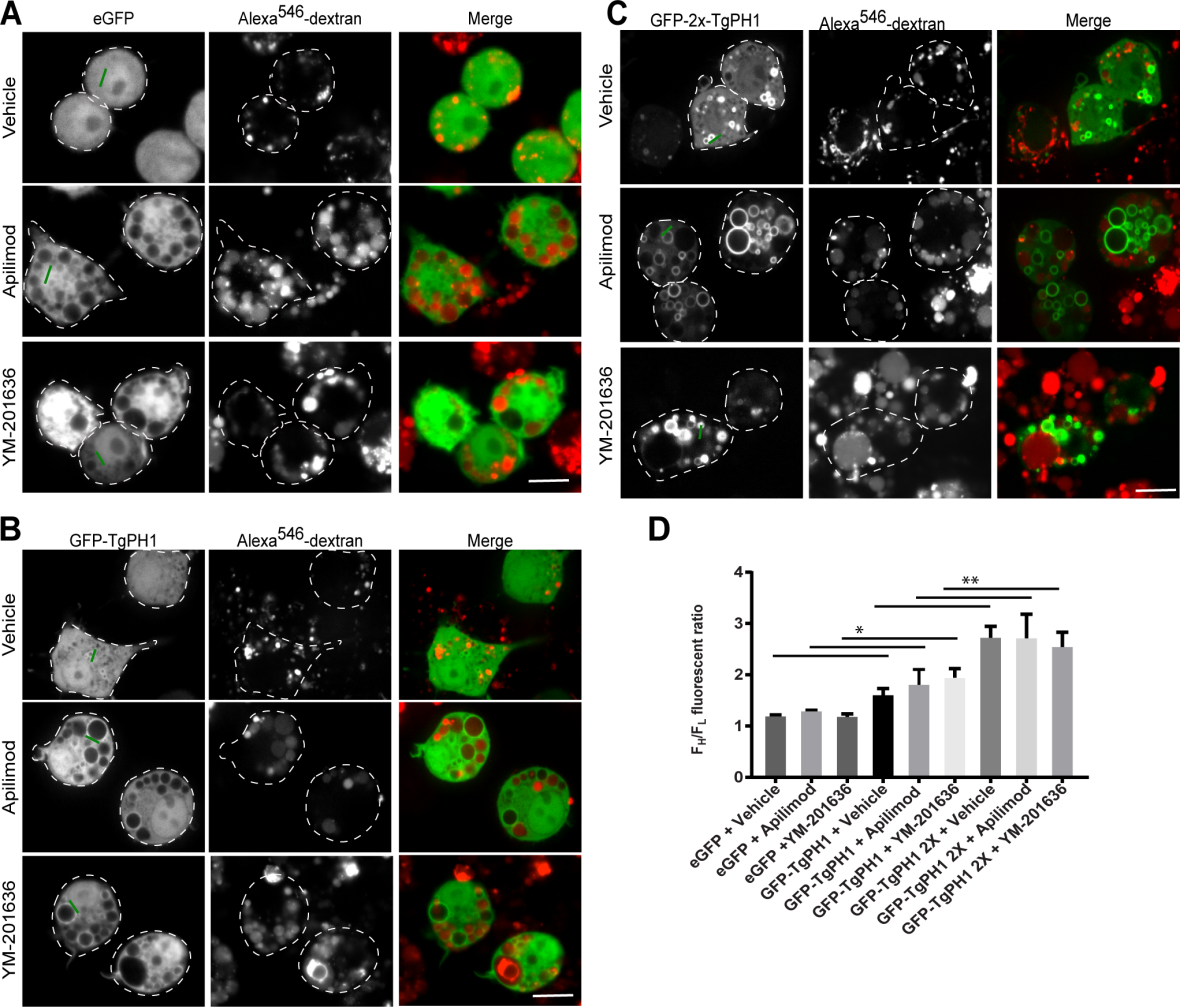
Subcellular distribution of GFP-fused TgPH1 proteins in PIKfyve-inhibited RAW cells. RAW macrophages were transfected with GFP (A), GFP-TgPH1 (B), or GFP-2x-TgPH1 (C) and lysosomes were labelled with Alexa^546^-conjugated dextran as described in Methods. Cells were then left untreated (control) or exposed to 20 nM apilimod or 100 nM YM201636 for 1 h to inhibit PIKfyve and induce vacuolation. Cells were imaged live using spinning disc confocal microscopy. GFP-2x-TgPH1 displayed strong membrane association that was retained in PIKfyve-inhibited conditions, while GFP-TgPH1 displayed weaker membrane association and GFP was cytosolic under all treatments employed. Green line exemplifies line arrangements used to quantify F^H^/F_L_ fluorescence ratio. D. Quantification of GFP, GFP-TgPH1 and GFP-2x-TgPH1 membrane association. Data shown are mean F^H^/F_L_ ± SD from N = 3 from 25–40 cells per experiment per condition. Using a one-way ANOVA and Tukey's post-hoc test, there was significant difference between the F^H^/F_L_ for GFP and GFP-TgPH1 for each respective condition (* p<0.05). In addition, there was a significant difference between the F^H^/F_L_ for GFP-TgPH1 and GFP-2x-TgPH1 (** p<0.01). Scale bar represent 10 μm.

**Fig 2:**
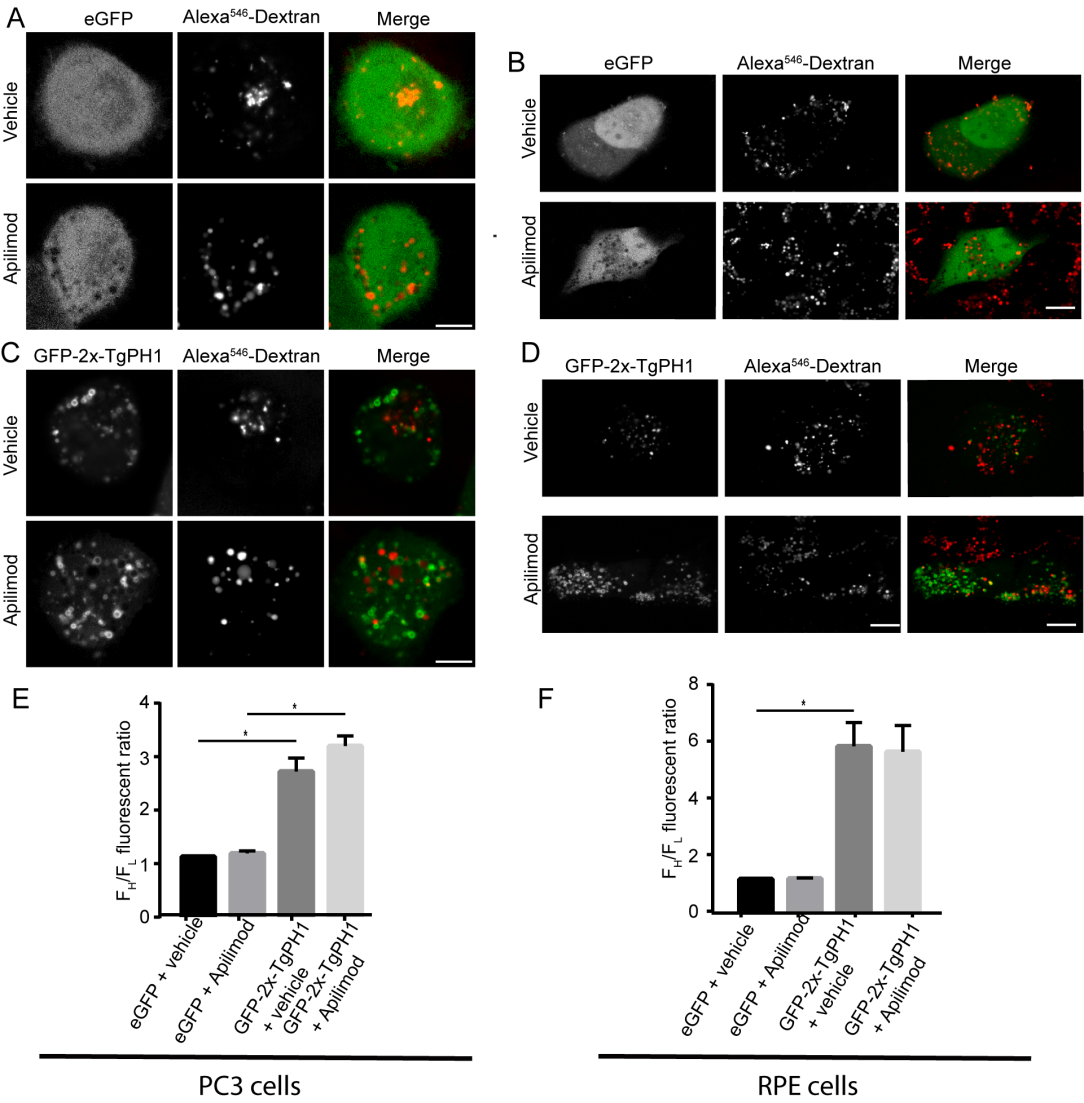
GFP-2x-TgPH1 retains membrane association in several mammalian cells inhibited for PIKfyve. Human PC3 cells and RPE cells were transfected with GFP or GFP-2x-TgPH1, labelled with fluorescent dextran to identify lysosomes and then treated with vehicle or apilimod to inhibit PIKfyve for 1 h, followed by spinning disc confocal imaging. A, B. PC3 cells (A) and RPE (B) expressing GFP treated with vehicle or apilimod. In all cases, GFP appeared cytosolic. C, D: PC3 cells (C) and RPE cells (D) expressing GFP-2x-TgPH1 treated with vehicle or apilimod. In all cases, GFP-2x-TgPH1 retained membrane association. E, F. Quantification of GFP and GFP-2x-TgPH1 membrane association in PC3 (E) and RPE cells (F). Data shown are mean F^H^/F_L_ ± STD from N = 3 from 25–40 cells per experiment per condition. Using a one-way ANOVA and Tukey's post-hoc test, there was a significance difference in the FH/FL for GFP versus GFP-2x-TgPH1 for the respective cells and treatments (* p<0.01) but there was no difference in the membrane distribution of GFP-2x-TgPH1 between vehicle and apilimod in PC3 or RPE cells. Scale bar represent 10 μm.

First, we used eGFP as a control for cytosolic-distributed proteins. As expected, eGFP was cytosolic in untreated RAW cells and those exposed to apilimod or YM201636, as reflected by an F^H^/F_L_ value of ~1 across all treatments (Fig 1A and 1D). In comparison, GFP-TgPH1 and GFP-2x-TgPH1 associated with membranes, albeit GFP-TgPH1 was significantly weaker than GFP-2x-TgPH1 (Fig 1B–1D). Importantly, GFP-TgPH1 and GFP-2x-TgPH1 both retained membrane association in RAW cells treated with either apilimod or YM201636 (Fig 1B–1D). As evidence of drug activity, apilimod and YM201636 treatments caused vacuolation, as expected. Interestingly, both GFP-TgPH1 and GFP-2x-TgPH1 co-localized poorly with dextran-labelled structures in control, suggesting that they did not localize to lysosomes (Fig 1). To ensure that these observations were not restricted to RAW macrophages, we expressed GFP-2x-TgPH1 in PC3 and RPE cells exposed to vehicle or apilimod. We obtained very similar results as in RAW cells, where GFP-2x-TgPH1 retained membrane association in vehicle-treated and apilimod-treated cells and poorly co-localized with dextran-labelled structures (Fig 2).

**Fig 3:**
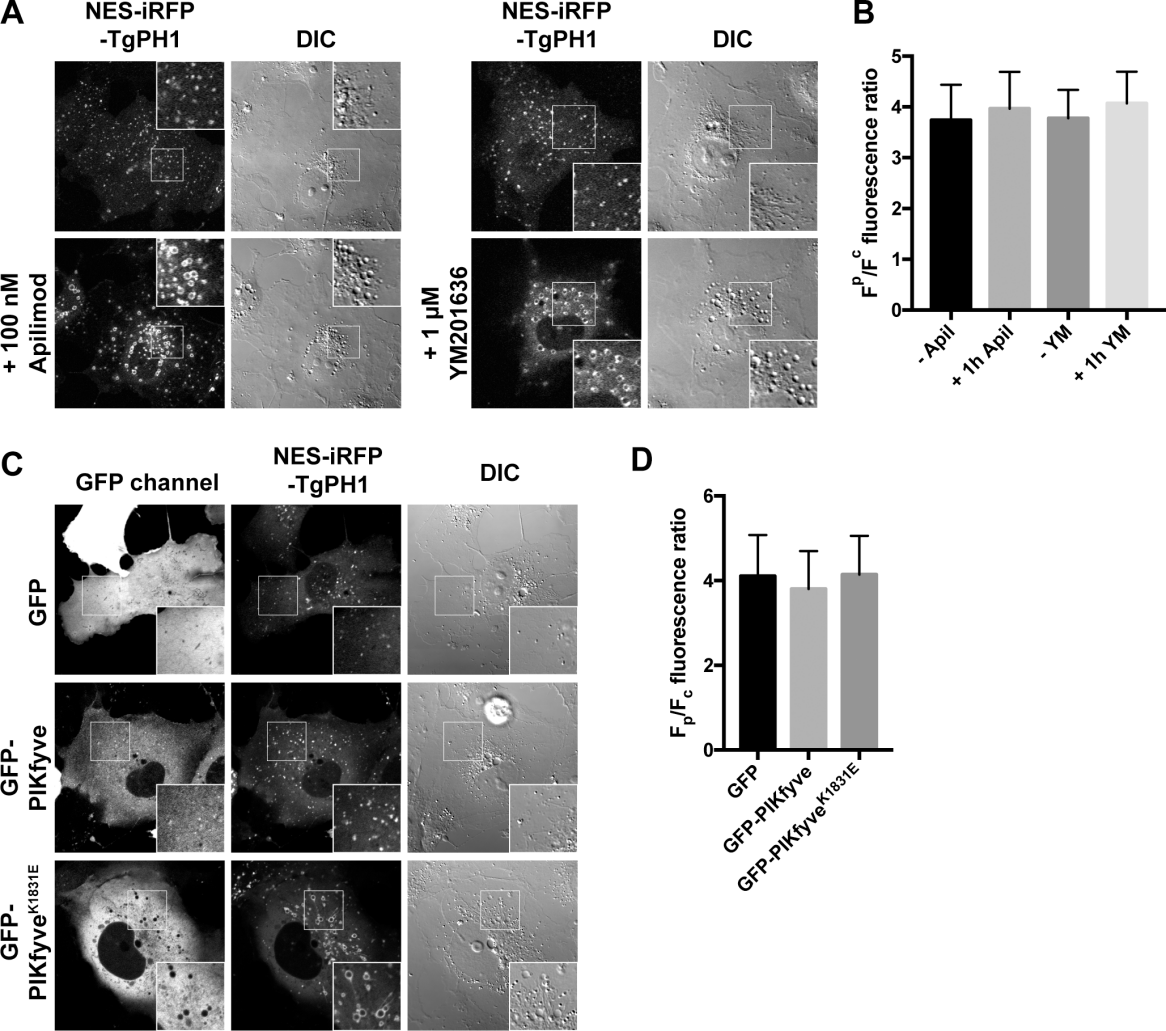
TgPH1 retains membrane association in genetically-impaired PIKfyve cells. A. Live-cell imaging of Cos-7 cells expressing NES-iRFP-TgPH1 treated with vehicle alone or with 100 nM apilimod or 1 μM YM201636 for 1 h; the drugs cause a characteristic appearance of swollen vacuoles, some of which labelled with the TgPH1 probes. (B) Quantification (means ± SD of 38 ≤ n ≤ 40 cells) of TgPH localization on vesicular structures in cells expressed as F_p_/F_c_, the ratio of intensity in bright punctate objects to mean fluorescence of the whole cell; there is no decrease in membrane association of TgPH1 in COS-7 cells after PIKfyve inhibition. (C) COS-7 cells imaged as in A whilst co-expressing either GFP, GFP-PIKfyve or its dominant-negative K1831E mutant. The latter causes the appearance of swollen vacuoles due to defective PtdIns(3,5)P_2_ synthesis, yet there is no effect on TgPH1 membrane association, as quantified in (D). D. Data shown is the mean ± SD of 90 cells per condition. Inset are zoomed areas (400 μm^2^ delineated by the box in each panel. Corresponding differential interference contrast (DIC) are also shown.

To complement these findings with a non-pharmacological approach, we co-expressed dominant-negative PIKfyve^K1831E^ and iRFP^NES^-TgPH1 in Cos-7 cells; we used Cos-7 cells whenever we needed to achieve high co-transfection rates concurrent with high-expression of target proteins. First, we showed that Cos-7 cells treated with apilimod or YM201636 retained membrane association of iRFP^NES^-TgPH1, despite triggering vacuolation (Fig 3A and 3B). Second, and more importantly, iRFP^NES^-TgPH1 associated with membranes in Cos-7 cells irrespective of whether they expressed GFP, GFP-PIKfyve or GFP-PIKfyve^K1821E^, even though GFP-PIKfyve^K1821E^ expression caused vacuolation (Fig 3C and 3D). Collectively, these data suggest that TgPH1 does not depend on PIKfyve activity for membrane association in mammalian cells, indicating that TgPH1 is not a viable probe for PtdIns(3,5)P_2_ in these cells.

### TgPH1 becomes cytosolic in Vps34 inhibited cells

**Fig 4:**
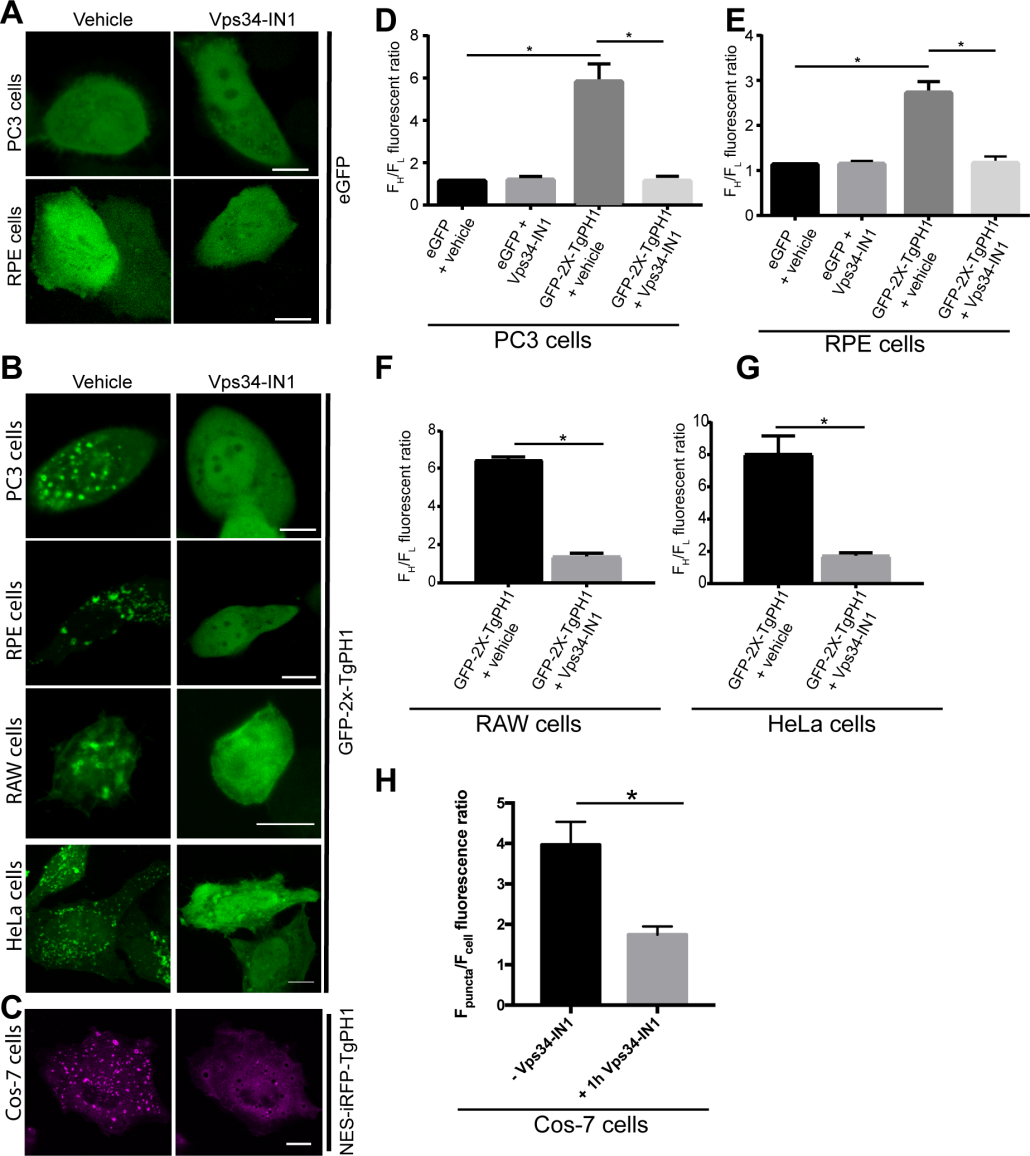
Inhibition of Class III PtdIns 3-Kinase Vps34 displaces TgPH1 domain from membranes. A. PC3 and RPE cells expressing eGFP and treated with vehicle or 1 μM VPS34-IN1 for 1 h to block Vps34 activity. B. PC3, RPE, HeLa and RAW macrophages expressing GFP-2x-TgPH and treated with vehicle (control) or 1 μM VPS34-IN1 for 1 h. For A and B, cells were fixed and imaged by spinning disc confocal microscopy. C. Cos-7 cells expressing NES-iRFP-TgPH treated with vehicle or 1 μM Vps34-IN1 for 1 h. Cells were imaged by laser scanning confocal microscopy. For B and C, control cells displayed chimeric TgPH1 proteins on punctate structures, while cells treated with Vps34-IN1 exhibit mostly cytosolic TgPH1 distribution. D-G: The proportion of GFP-2x-TgPH1 associated with membranes relative to the cytosol was estimated by quantifying F^H^/F_L_ fluorescence ratio as described in Methods using a 10-pixel wide and 30-40-pixel long line. H. The proportion of NES-iRFP-TgPH1 in the membrane versus cytosol was assessed by quantifying F_p_/F_c_ as described in Methods. In all cases, these data quantitatively show that GFP-2x-TgPH1 and NES-iRFP-TgPH1 become cytosolic in VPS34-IN1-treated cells in a variety of cell lines. Error bars represent standard error of the mean derived from analyzing at least 20 cells per condition across N = 3 independent experiments. * p<0.05 against respective controls using Student’s t-test for F, G and H or using one-way ANOVA and Tukey’s test for D and E. Scale bars represent 10 μm.

We then speculated that TgPH1 may bind to membranes in mammalian cells via PtdIns(3)P instead. To test this, we transfected RAW, HeLa, PC3, RPE and Cos-7 cells with TgPH1 probes and treated cells with DMSO or VPS34-IN1, a selective inhibitor of Vps34 Class III PI3K. VPS34-IN1 was previously shown to deplete cells of PtdIns(3)P and disperse PtdIns(3)P probes from endosomes into the cytosol [26]. As before, we quantified the membrane to cytosol distribution using F^H^/F_L_ ratio of GFP-fusion proteins. First, we verified that VPS34-IN1 did not alter eGFP distribution in various cell types as a control; as shown for PC3 and RPE cells, GFP remained cytosolic in both vehicle and VPS34-IN1-treated cells (Fig 4A, quantified in Fig 4C and 4E). In comparison, and as indicated above, GFP-2x-TgPH1 exhibited membrane-bound distribution in RAW, HeLa, PC3 and RPE cells exposed to vehicle alone (Fig 4B, quantified in Fig 4D–4G). By contrast, all cell types treated with VPS34-IN1 exhibited mostly cytosolic GFP-2x-TgPH1 distribution (Fig 4B and 4D–4G). To reflect the experiments in Fig 3, we also transfected Cos-7 cells with NES-iRFP-TgPH1 and examined its localization with vehicle or VPS34-IN1-treated cells. As with the other cell types, Cos-7 cells exposed to VPS34-IN1 exhibited mostly cytosolic iRFP^NES^-TgPH1, while iRFP^NES^-TgPH1 was membrane-bound in vehicle-treated Cos-7 cells (Fig 4C and 4H). Overall, these data indicate that TgPH1 associates to intracellular membranes in a PtdIns(3)P-dependent manner in mammalian cells.

### Inducible depletion of PtdIns(3)P dissociates TgPH1 from membranes

**Fig 5:**
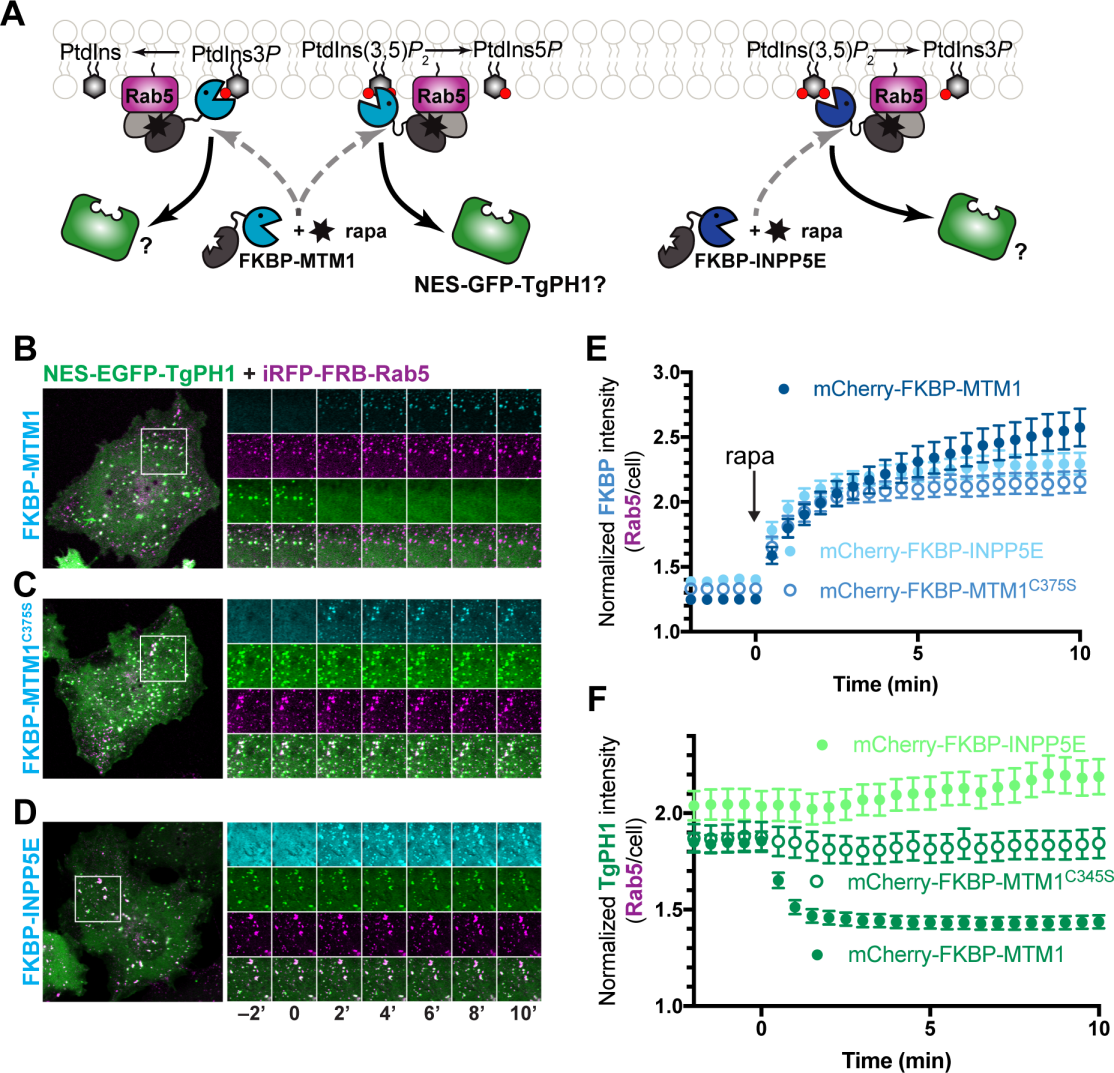
Depletion of PtdIns(3)P but not PtdIns(3,5)P_2_ causes TgPH1 to dissociate from Rab5-positive membranes in COS-7 cells. A. Schematic of the rapamycin-induced dimerization system and the enzymatic activities of the FKBP-conjugated phosphatases. B, C. Time-lapse imaging of COS-7 cells expressing iRFP-FRB-Rab5 (magenta), mCherry-FKBP fused to the indicated enzyme (cyan) and eGFP^NES^-TgPH1 (grayscale). B. The time-lapse shows the recruitment of the fused phosphatase (cyan) to Rab5-containing puncta (magenta) before and after the addition of 1 μM rapamycin, marked by time 0. C. A time-lapse series of the same cells showing the dynamics of eGFP^NES^ -TgPH1 before and after rapamycin. Images were acquired at 2 min intervals. The graphs at right show the normalized intensity at Rab5-positive membranes relative to the whole cell for FKBP-tagged enzymes (B) and eGFP^NES^-TgPH1 (C). Data are means ±SEM of 41 (MTM1), 23 (C375S) or 27 (INPP5E) cells from 3 or 4 independent experiments. * p<0.05 against control using ANOVA and Tukey's test.

We next complemented the pharmacological depletion of PtdIns(3)P and PtdIns(3,5)P_2_ using 3- and 5-phosphatases genetically engineered to associate with Rab5-positive early endosomes with a rapamycin-inducible dimerization system, previously characterized [42–44,48]. We targeted Rab5-decorated endosomes because they are thought to be enriched in PtdIns(3)P. Briefly, FRB-fusion of Rab5 is co-expressed with an FKBP-fusion of one of two lipid phosphatases: the MTM1 lipid phosphatase, which removes 3-phosphate from PtdIns(3)P and PtdIns(3,5)P_2_, or with the INPP5E lipid phosphatase, which was shown to remove 5-phosphate from several lipids including PtdIns(3,5)P_2_ [48–52](Fig 5A). As a control, we also included the catalytic-dead MTM1^C375S^ mutant. First, we show that MTM1, MTM1^C375S^ and INPP5E are initially cytosolic but associate with iRFP-Rab5-labelled structures within 5 min of rapamycin addition (Fig 5B–5D and 5E shows the quantification of MTM1 and INPP4E co-localization with Rab5). We then tracked the dynamics of eGFP^NES^-TgPH1 in these cells co-expressing the inducible dimerization constructs. We note that before adding rapamycin to cells, eGFP^NES^-TgPH1 localized to iRFP-FRB-Rab5 puncta, showing that TgPH1 associated with early endosomes. However, upon addition of rapamycin to cells co-expressing FKBP-MTM1 fusion, there was a dramatic divestment of eGFP^NES^-TgPH1 from puncta (Fig 5B and 5F) that correlated with the recruitment of MTM1 to Rab5-labelled endosomes (Fig 5B and 5F). Expression of MTM1^C375S^, a phosphatase-dead mutant, and addition of rapamycin failed to dissociate eGFP^NES^-TgPH1 from Rab5-labelled puncta showing that this required degradation of PtdIns(3)P (Fig 5C and 5F). In addition, expression of INPP5E also failed to delocalize eGFP^NES^-TgPH1 from Rab5 structures, suggesting that TgPH1 does not associate with membranes via PtdIns(3,5)P_2_ (Fig 5D and 5F), and consistent with our experiments with PIKfyve suppression. Collectively, these data are consistent with PtdIns(3)P, but not PtdIns(3,5)P_2_, as the lipid that recruits TgPH1 to membranes in mammalian cells.

### TgPH1 co-localizes with other PtdIns(3)P fluorescent probes

**Fig 6:**
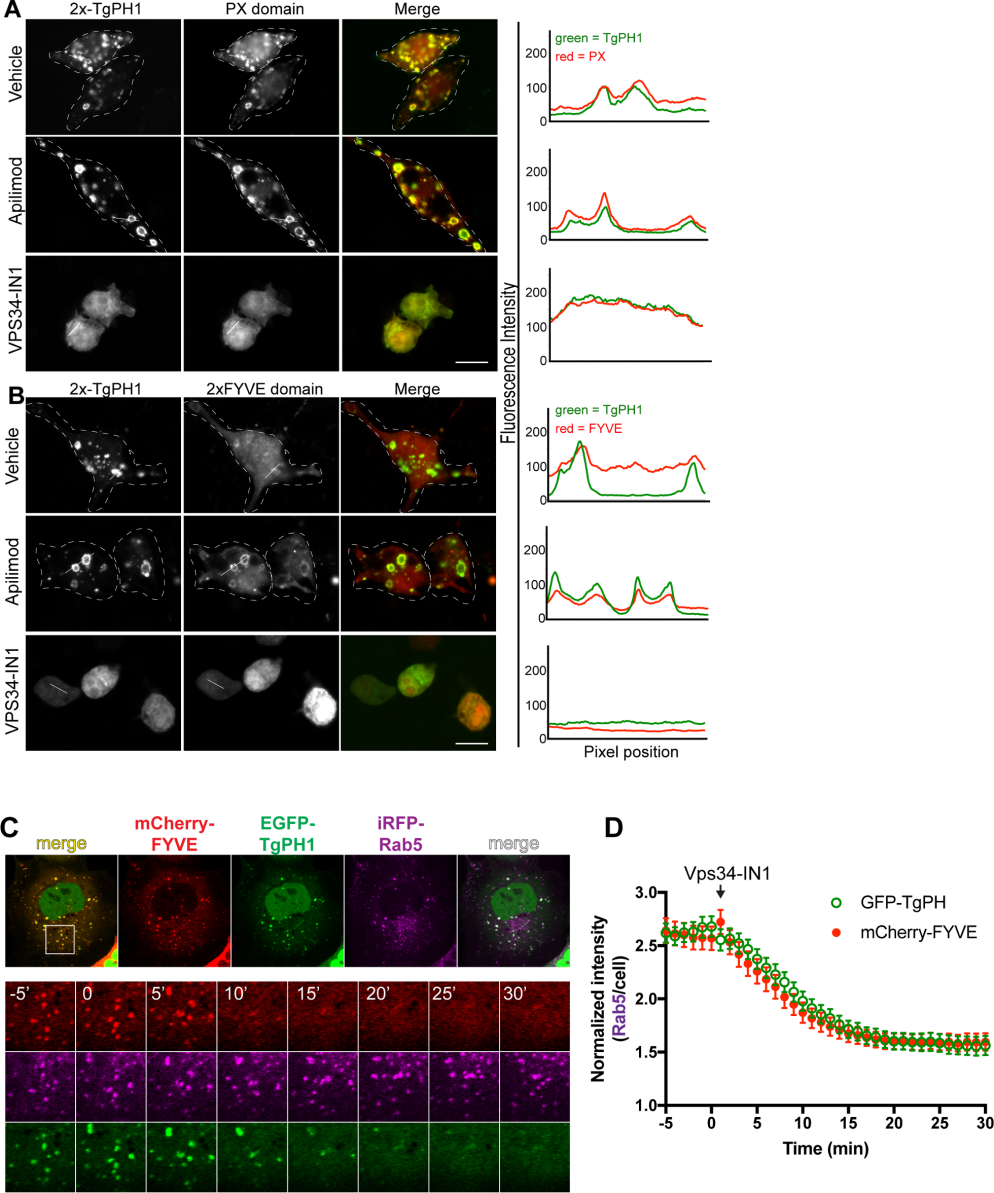
GFP-2x-TgPH1 strongly co-localize with other PtdIns(3)P probes. RAW cells were co-transfected with GFP-2x-TgPH1 and p40PX-mCherry (A) or with 2FYVE-mRFP (B). Cells were left untreated (control) or treated with 20 nM apilimod or 1 μM VPS34-IN1 for 1 h. Lines drawn across punctate structures in both green and red channels show coincident fluorescence intensity plots for TgPH1 chimera with either p40PX-mCherry or 2FYVE-mRFP. Images are representative of cells from N = 3 independent experiments using at least 25 cells per condition per experiment. C. Cos-7 cells co-expressing GFP^NES^-TgPH1, mCherry-FYVE and iRFP-Rab5 to mark early endosomes and imaged by time-lapse confocal microscopy during treatment with 1 μM Vps34-IN1. Note the near identical enrichment of both PtdIns(3) P probes at Rab5-positive membranes before addition of Vps34-IN1 at -5 and 0 min. This is followed by a nearly coincidental displacement of GFP^NES^-TgPH1 and mCherry-FYVE within 10 min of Vps34-IN1 addition. Inset region = 16 μm x 16 μm. Time shown is in minutes, where 0 min marks the addition of Vps34-IN1. D. Graph shows the fluorescence intensity of both TgPH1 and FYVE at Rab5-positive membranes (normalized to whole cell intensity) as a function of time, with Vps34-IN1 added at time 0; data are means ± SEM of 32 cells per group.

Our data suggest that TgPH1 is a potential novel reporter for PtdIns(3)P in mammalian cells that is of non-mammalian origin and is distinct from the most commonly employed PtdIns(3)P-binding probes, which consist of either a FYVE or PX domain [15,22,29,53]. To better assess this possibility, we compared the behaviour of TgPH1 constructs with well-characterized PtdIns(3)P reporters. First, we characterized the behaviour of GFP-2x-TgPH1 co-expressed with PX-mCherry or with mRFP-2FYVE in RAW cells. In both cases, GFP-2x-TgPH1 co-localized strongly with PX-mCherry (Fig 6A) and 2FYVE-RFP (Fig 6B). This was made more apparent when cells were treated with apilimod, which enlarged structures labelled with all three probes (Fig 6A and 6B). In comparison, all three chimeric proteins became cytosolic upon exposure with VPS34-IN1 to deplete cells of PtdIns(3)P (Fig 6A and 6B). Second, to better assess the spatio-temporal dynamics of TgPH1 and a FYVE domain probe, we co-expressed GFP^NES^-TgPH1 with mCherry-FYVE and iRFP-Rab5 in Cos-7 cells to concomitantly tracked their behaviour in live cells. We observed that all three proteins significantly co-localized in Cos-7 cells, consistent with TgPH1 and FYVE domains reporting PtdIns(3)P on early endosomes (Fig 6C; -5 min and 0 min prior to addition of Vps34-IN1). We then added Vps34-IN1 to block PtdIns(3)P and quantified the distribution of FYVE and TgPH1 to Rab5-labelled structures. Indeed, both FYVE and TgPH1 lost most of their punctate distribution within 10 min of arresting PtdIns(3)P synthesis (Fig 6C). Overall, GFP-2x-TgPH1 co-localizes with established PtdIns(3)P biosensors and represents a complementary biosensor for PtdIns(3)P.

## Discussion

In this study, we expressed and characterized the PH domain from *T*. *gondii* PH-domain containing protein-1 (TgPH1). This domain was previously suggested to bind to PtdIns(3,5)P_2_ in *T*. *gondii* using *in vitro* assays [41]. We thus postulated that TgPH1 could report PtdIns(3,5)P_2_ in mammalian cells. However, our data negate this hypothesis. Instead, we provide evidence that TgPH1 expressed in various mammalian cells either as a single or as a tandem-fusion, associates with membranes in a PtdIns(3)P-dependent manner. This conclusion is supported by pharmacological and genetically-encoded depletion system of PtdIns(3)P and PtdIns(3,5)P_2_. Specifically, TgPH1 dissociated from membranes only in cells treated with inhibitors of Vps34, but not of PIKfyve. In addition, while induced targeting of INPP5E, a 5-phosphatase that has been shown to deplete PtdIns(3,5)P_2_ but not PtdIns(3)P, did not displace TgPH1 from membranes. However, targeting of MTM1, which eliminates PtdIns(3)P divested TgPH1 from Rab5-positive membranes. We have not entirely excluded that TgPH1 may associate with membranes by binding to either PtdIns(3)P or PtdIns(3,5)P_2_. Indeed, TgPH1 bound both PtdIns(3)P and PtdIns(3,5)P_2_ when using lipid blots [41]. However, if binding to PtdIns(3)P and PtdIns(3,5)P_2_ contributed to recruitment of TgPH1 to membranes, we should have observed a reduction in F^H^/F_L_ or co-localization with Rab5 in cells inhibited for PIKfyve; since this was not the case, we argue that any interaction between TgPH1 and PtdIns(3,5)P_2_ is an insignificant contributor to membrane association compared to TgPH1-binding to PtdIns(3)P. Overall, we can confidently conclude that TgPH1 is not a viable biosensor for PtdIns(3,5)P_2_ in mammalian cells. Instead, we propose that TgPH1 may serve as a complementary reporter of PtdIns(3)P in mammalian cells.

Having complementary reporters for PtdIns(3)P is an important consideration since PtdInsP-binding domains carry caveats when employed to investigate PtdInsP distribution and dynamics [8,18]. Even well-characterized probes like the PH domains of FAPP1, PLCδ and Akt, which respectively track PtdIns(4)P, PtdIns(4,5)P_2_, PtdIns(3,4,5)P_3_ carry caveats [8,9,11]. For example, the PH domain of PLCδ1, while commonly employed as a reporter for PtdIns(4,5)P_2_ also binds with higher affinity to soluble inositol-1,4,5-bisphosphate (IP_3_) [54,55]. In another example, the PH domain of FAPP1 reports Golgi-associated PtdIns(4)P because it also binds to the Golgi-associated Arf1 GTPase [20,21]. Alternative probes for these lipids respectively include the PH domain of Tubby1 that binds PtdIns(4,5)P_2_ but not IP_3_ and the *Legionella*-derived P4M, which detects multiple pools of PtdIns(4)P including on late endosomes [42,54,55]. Similarly, the PX and FYVE-based probes for PtdIns(3)P have potential flaws. For example, the FYVE domain of EEA1 carried an adjacent motif that associated with GTP-bound Rab5 [15,23,56]. In fact, in yeast, PtdIns(3)P was initially thought to be restricted to early endosomes only, but subsequent use of other probes including FYVE domain of Fab1 show that PtdIns(3)P exists in vacuoles [57]. Thus, it is important to develop complementary probes for PtdIns(3)P.

TgPH1 has several advantages as a complementary tool to study PtdIns(3)P. First, the majority of PtdIns(3)P probes tend to be FYVE or PX domain based [8,9,15,18,29]. TgPH1 is wholly distinct as a PH domain. Second, TgPH1 is unique among other PH domains since the vast majority of PtdInsP-binding PH domains bind to PtdIns(4)P, PtdIns(3,4)P_2_, PtdIns(4,5)P_2_, and PtdIns(3,4,5)P_3_, not PtdIns(3)P. Lastly, TgPH1 is not mammalian-derived and may reduce the chances that it reports sub-pools of PtdIns(3)P due to co-incident binding of an endogenous protein ligand [8,11,18,19]. Overall, while TgPH1 did not fulfill our original intention of serving as a complementary tool for investigating PtdIns(3,5)P_2_, we propose that it may be a useful complementary tool to investigate the location, sub-domain distribution and dynamics of PtdIns(3)P in mammalian cells.
