# Correction: The PH domain from the *Toxoplasma gondii* PH-containing protein-1 (TgPH1) serves as an ectopic reporter of phosphatidylinositol 3-phosphate in mammalian cells

**DOI:** 10.1371/journal.pone.0201800

**Published:** 2018-07-31

**Authors:** Krishna Chintaluri, Brady D. Goulden, Camilyn Clemenza, Golam Saffi, Emily Miraglia, Gerald R. V. Hammond, Roberto J. Botelho

The third author’s name is spelled incorrectly. The correct name is: Camilyn Clemenza. The correct citation is: Chintaluri K, Goulden BD, Clemenza C, Saffi G, Miraglia E, Hammond GRV, et al. (2018) The PH domain from the *Toxoplasma gondii* PH-containing protein-1 (TgPH1) serves as an ectopic reporter of phosphatidylinositol 3-phosphate in mammalian cells. PLoS ONE 13(6): e0198454. https://doi.org/10.1371/journal.pone.0198454
